# Water content estimation of conifer needles using leaf-level hyperspectral data

**DOI:** 10.3389/fpls.2024.1428212

**Published:** 2024-09-06

**Authors:** Yuan Zhang, Anzhi Wang, Jiaxin Li, Jiabing Wu

**Affiliations:** ^1^ CAS Key Laboratory of Forest Ecology and Silviculture, Institute of Applied Ecology, Chinese Academy of Sciences, Shenyang, China; ^2^ University of Chinese Academy of Sciences, Beijing, China

**Keywords:** gymnosperms, water content, hyperspectral data, water indices, partial least squares regression

## Abstract

Water is a crucial component for plant growth and survival. Accurately estimating and simulating plant water content can help us promptly monitor the physiological status and stress response of vegetation. In this study, we constructed water loss curves for three types of conifers with morphologically different needles, then evaluated the applicability of 12 commonly used water indices, and finally explored leaf water content estimation from hyperspectral data for needles with various morphology. The results showed that the rate of water loss of Olgan larch is approximately 8 times higher than that of Chinese fir pine and 21 times that of Korean pine. The reflectance changes were most significant in the near infrared region (NIR, 780-1300 nm) and the short-wave infrared region (SWIR, 1300–2500 nm). The water sensitive bands for conifer needles were mainly concentrated in the SWIR region. The water indices were suitable for estimating the water content of a single type of conifer needles. The partial least squares regression (PLSR) model is effective for the water content estimation of all three morphologies of conifer needles, demonstrating that the hyperspectral PLSR model is a promising tool for estimating needles water content.

## 1 Introduction

Leaves play a crucial role in plant photosynthesis, and carry essential physiological functions. During photosynthesis, CO_2_ enters the leaf tissue through the stomata, and the leaves constantly lose water in the process. Under severe drought stress, the relative water content of leaves decreases progressively, which reduces stomatal conductance, slows CO_2_ assimilation, and eventually stops photosynthesis (Lawlor, 2002). This indicates that the photosynthetic performance is highly dependent on the stable and suitable water status of leaves (Xiong and Nadal, 2020). In comparison with whole-leaf nitrogen and phosphorus masses, leaf water mass is a better predictor of whole-leaf photosynthesis (Wang et al., 2022). Therefore, accurately estimating leaf water content is crucial for detecting drought stress and monitoring plant health status.

Contactless approaches, such as electromagnetic spectroscopy and radar, are the primary techniques used to estimate the water content of individual leaves (Quemada et al., 2021). Over the past decade, portable spectrometers have emerged as a popular electromagnetic spectroscopy tool. Its hyperspectral sensors can divide the spectrum into dozens to hundreds of narrow bands, enabling the precise measurement of physiological and biochemical parameters in plants. The biochemistry and internal structure of leaves control the optical signatures observed (Serbin and Townsend, 2020). Leaf water content (Naik et al., 2020), chlorophyll concentration (Ling et al., 2019), and nutrients (Liu et al., 2023) are the three key parameters of interest. In terms of theory, the absorbed bands of chlorophyll are concentrated in the visible bands (VIS, 380–780 nm), while the other two class parameters are concentrated in the short-wave infrared region (SWIR, 1300–2500 nm). However, most studies have focused on field crops (Khan et al., 2022), with limited attention given to tall trees, particularly conifers. An early study demonstrates that there is a critical link between the physiological characteristics of needles and spectral signatures in two co-occurring conifers (Stimson et al., 2005). And the spectral changes were detected earlier than observable in the field, when water stress occurred (Einzmann et al., 2021).

Conifers are non-porous trees (Gymnosperms) with conservative water use strategies, and their growth processes are highly sensitive to drought stress (Zlobin, 2022). They may be more vulnerable to extreme drought than broadleaved species under climate warming (Yuan et al., 2021). Different conifers have various foliage morphologies, such as linear-acicular leaves, and scale-like leaves. Most conifer needles have a cuticular wax and several narrow bands of stomatal lines. Conifers have rather low stomatal densities in comparison to broadleaf species and herbs (Xiong and Flexas, 2020), which allow to minimize water loss. Therefore, studying the simulation and estimation of water loss of conifer needles can help understand their coping strategies to drought stress. The objectives of this study were: 1) to propose the acquisition method of hyperspectral data according to three types of conifers with morphologically different needles; 2) to construct the water loss curve of needles, and analyze the reasons for the differences in water loss rate; 3) to determine the sensitive bands of leaf water, verify and analyze the applicability of commonly used water indices; and 4) to explore a hyperspectral estimation machine learning method for needles with various morphology.

## 2 Materials and methods

### 2.1 Study area

Our study area was located in the National Natural Reserve of Changbai Mountain (42°24′9″N, 128°05′45″E), Jilin Province, Northeast China. Changbai Mountain has typical temperate vertical vegetation zones, including a rich variety of tree species within its ecosystem. Three types of conifers were selected for this study, based on the characteristics of their respective needles (**Table 1**). The three conifers belong to Pinaceae plants, including the genera of *Pinus*, *Larix*, and *Abies*, respectively.

**Table 1:** Three conifers and the characteristics of these needles.

Species	Latin name	Characteristics of needles
Korean pine	*Pinus koraiensis* Siebold & Zucc.	Needles 5 per bundle, dark green, straight, almost triangular in cross section, 6-12 cm, stomatal lines 6-8 along each abaxial surface, blue-gray, vascular bundle 1, resin canals 3.
Olgan larch	*Larix olgensis* A. Henry	Leaves 1.5-2.5 cm × ca. 1 mm, keeled abaxially, stomatal lines 1-2 along each side of midvein on the upper surface, 2-5 on the lower surface.
Chinese fir pine	*Abies holophylla* Maxim.	Leaves pectinately arranged in 2 lateral sets, dark green and shining adaxially, linear, flattened, 2-4 cm × 1.5-2.5 mm, stomatal lines in 2 white bands abaxially, resin canals 2.

(Referred to www.worldfloraonline.org and www.iplant.cn).

### 2.2 Data acquisition

Mature older sun needles were collected from three types of conifers. Each type consists of five trees, and then the needles of each tree were divided into 3 groups (total 15 groups) for natural drying treatment in the laboratory (25°C, 40% RH), respectively. Different morphologies of conifer needles were set at different natural drying time intervals (**Table 2**) to obtain the actual weight and spectral reflectance. It should be noted that due to slow water loss of Korean pine, the needles color changes greatly after long-term water loss, so its water content is higher than 35%. Finally, the needles were dried at 65°C for 72 h to record the dry weight.

**Table 2:** Different natural drying time intervals of three morphologies of conifer needles.

Species	Drying time intervals
Korean pine	24h
Olgan larch	0, 1, 1, 2, 2, 2, 2, 2, 4h
Chinese fir pine	0, 4, 12, 16, 16, 16, 16, 16, 16h

**Figure 1:**
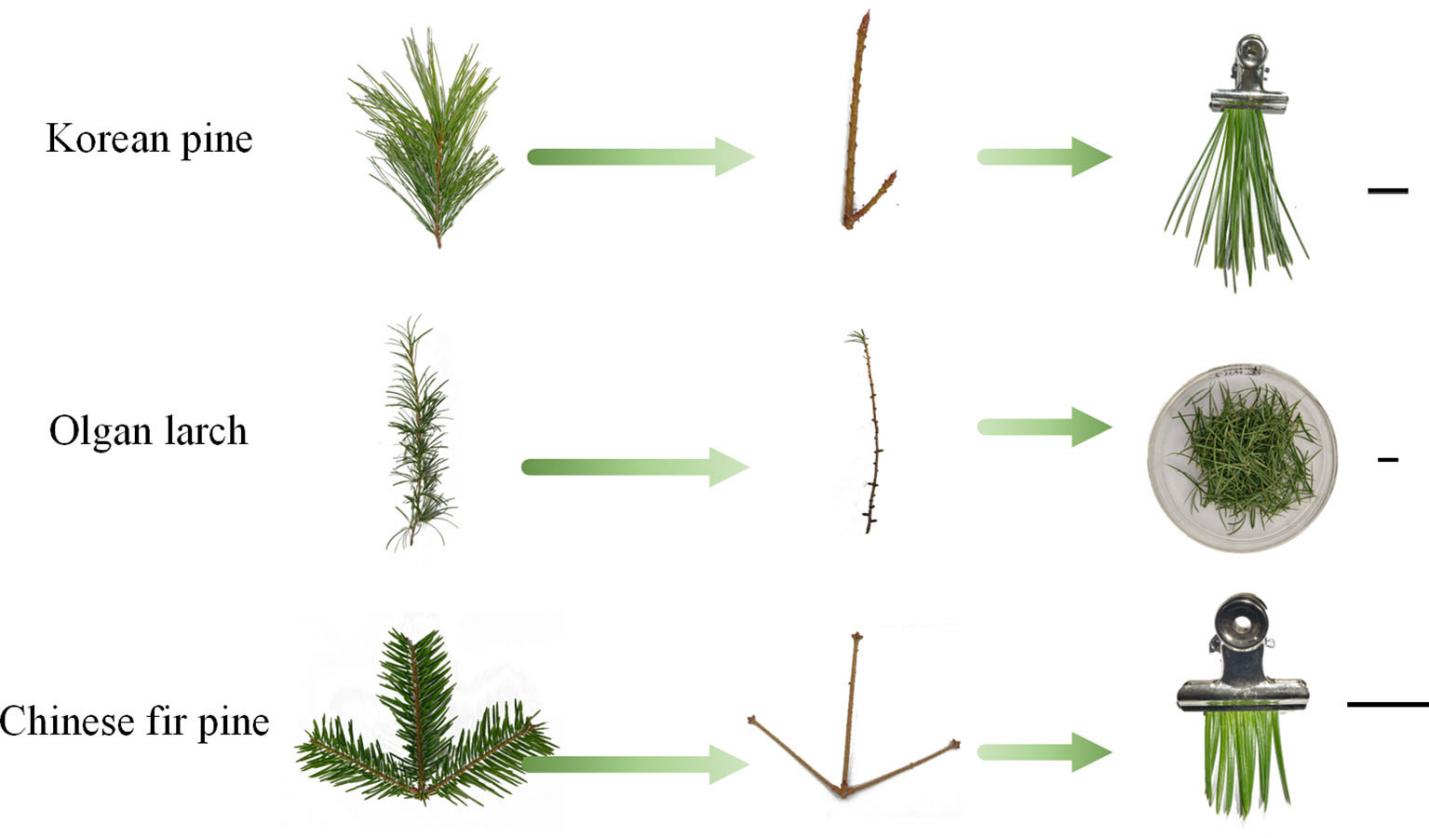
Needles were processed for spectral reflectance measurement. Each scale bar is 2.0 cm.

Spectral reflectance of the leaves was measured using an ASD FieldSpec4 device (Analytical Spectral Devices). Three sets of individual measurements were taken from different positions of the needles, with each set representing the mean value of ten measurements. A total of 1215 spectral reflectance data sets (15 groups, 9-time intervals, and 3 measurements) were recorded. The contact probe of ASD is not well-suited for directly measuring spectral reflectance of conifer needles, which have very narrow leaves. The usual method is to cut off the needles from the twigs and place on a plate for measuring (Einzmann et al., 2014; Hejtmanek et al., 2022). In this study, this method was used for Olgan larch. And put the needles in the culture dish as much as possible to avoid reflection interference from other materials in the background. For harder or longer needles, it’s challenging to fill the contact probe of the spectrometer when the needles are placed on a plate. To overcome this issue, we developed an acquisition method after multiple attempts: needles were plucked from the twigs, and collected by clamp (**Figure 1**), and then the spectral reflectance data were measured. Clamp can securely hold the needles. For each of the three conifers, about 8 bundles of needles, 2 grams of needles, and 12 needles were selected to fill the field of view of the contact probe, respectively. This new method ensures that the contact probe is fully covered by needles.

Weight was recorded using an electronic balance CPA225D (Sartorius) at 0.01 mg accuracy. Water content was calculated according to **Equation 1**.


(1) WC=(FW−DW)/DW

Notes: FW (fresh weight, g), DW (dry weight, g).

### 2.3 Water content fitting and estimation

The water content of the picked needles changed with time. To model the change in water content over time, and evaluate the rate of water loss, we used an exponential function.

Four methods are used to quantify the biophysical variables of vegetation from spectral data: parametric regression, nonparametric regression, physically based, and hybrid regression methods (Verrelst et al., 2019). In our study, the estimation of water content using hyperspectral data includes two parts: the water index method (parametric regression) and the machine learning method (hybrid regression methods). The latter data is divided into two parts: model training data (1/3) and validation data (2/3).

We identified 12 water indices through previous studies, including Water Index (WI) (Penuelas et al., 1997), Moisture Stress Index (MSI-1) (Hunt and Rock, 1989), Disease-Water Stress Index (DWSI) (Apan et al., 2004), Leaf Water Index (LWI) (Seelig et al., 2008), Moisture Stress Index (MSI-2) (Rock et al., 1986), Normalized Difference Water Index (NDWI) (Gao et al., 1996), Normalized Difference Infrared Index (NDII) (Hardisky et al., 1983), Normalized Heading Index (NHI) (Pimstein et al., 2009), NDWI-Hyperion (NDWI-Hyp) (Ustin et al., 2002), Double Difference Index (DDI) (Wang and Li, 2012), Floating Position Water Band Index (FPWBI) (Strachan et al., 2002), and Global Vegetation Moisture Index (GVMI) (Ceccato et al., 2002). These band indices are related to the water content of different parts of the plant, and a brief description can be found in **Supplementary Table S1**.

Partial Least Squares Regression (PLSR) is the more robust of multiple machine learning methods, and can be considered the optimal technique for predicting the water content of broad-leaved trees (Zhang et al., 2022). There is a best-practice guide to predicting plant traits using PLSR (Burnett et al., 2021). In this study, the achievement of PLSR using R language with the package “pls” (Mevik and Wehrens, 2007). Segmented cross-validation method is used in PLSR. And 10 principal components were identified by standard error based on cross-validation residuals.

### 2.4 Analysis approaches

An exponential function class (y=a*e^-b*x^+c) was used to evaluate the water loss of needles. The higher the coefficient b, the rapider the rate of water loss.

The water sensitive bands were determined by Pearson correlation coefficient (R). The inversion results of water content based on hyperspectral data were evaluated by coefficient of determination (R^2^), and root mean square error (RMSE).

## 3 Results

### 3.1 Water loss and curve fitting of needles

**Figure 2:**
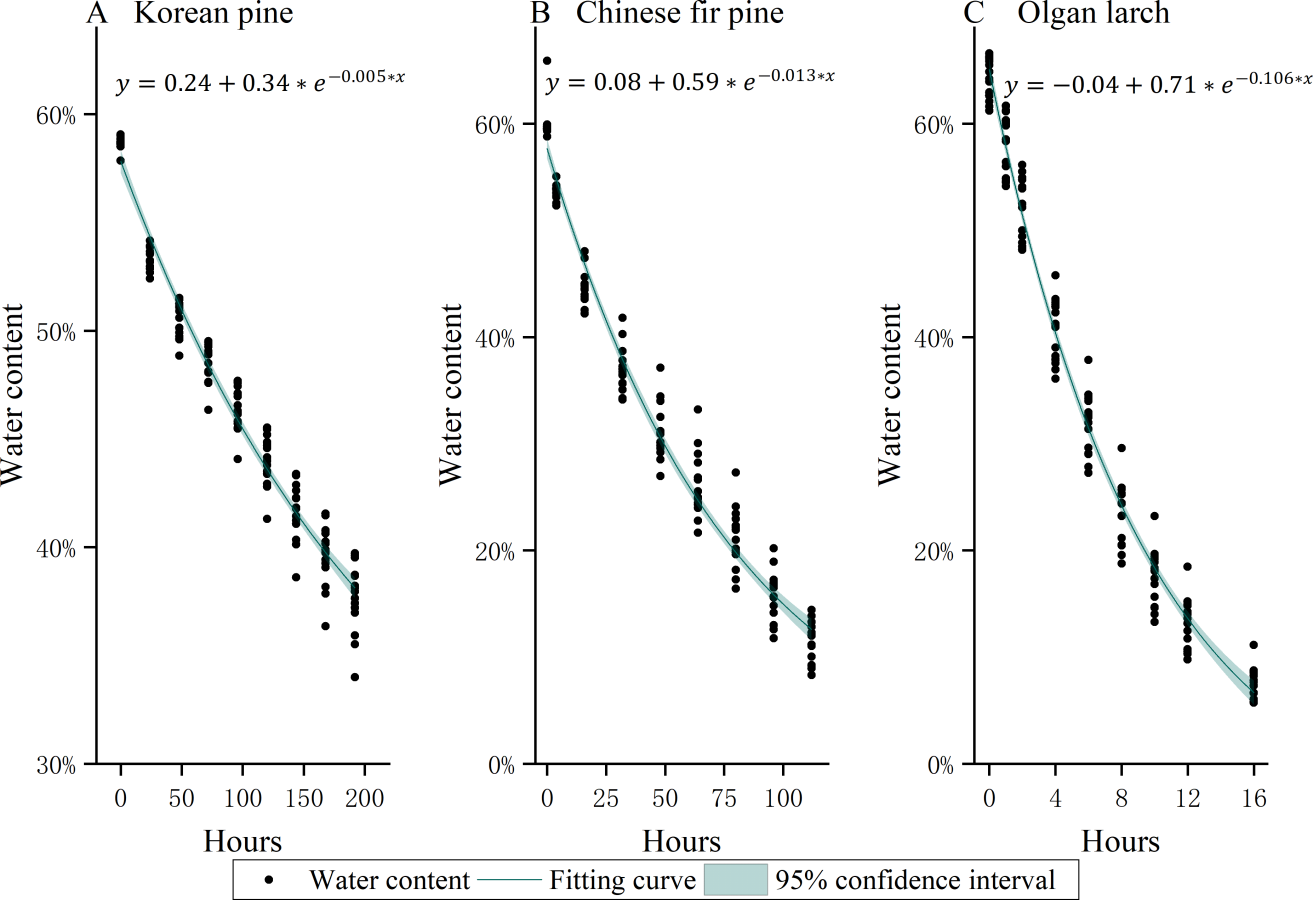
The water loss curve of leaves of three morphologies of conifer needles **(A)** Korean pine, **(B)** Chinese fir pine, and **(C)** Olgan larch.

The water content of needles decreases over time, after being picked from the trees (**Figure 2**). The rate of water loss reduction is fastest at the initial stage (the first two-time intervals of natural drying treatment), and then it becomes slower and slower.

The water loss of needles follows an exponential function distribution, and varies among different morphologies of conifer needles. Chinese fir pine has the fastest rate of water loss, followed by Olgan larch, while Korean pine has the slowest rate. The rate of water loss of Olgan larch is approximately 8 times higher than that of Chinese fir pine and 21 times that of Korean pine.

### 3.2 Changes of spectral reflectance

**Figure 3:**
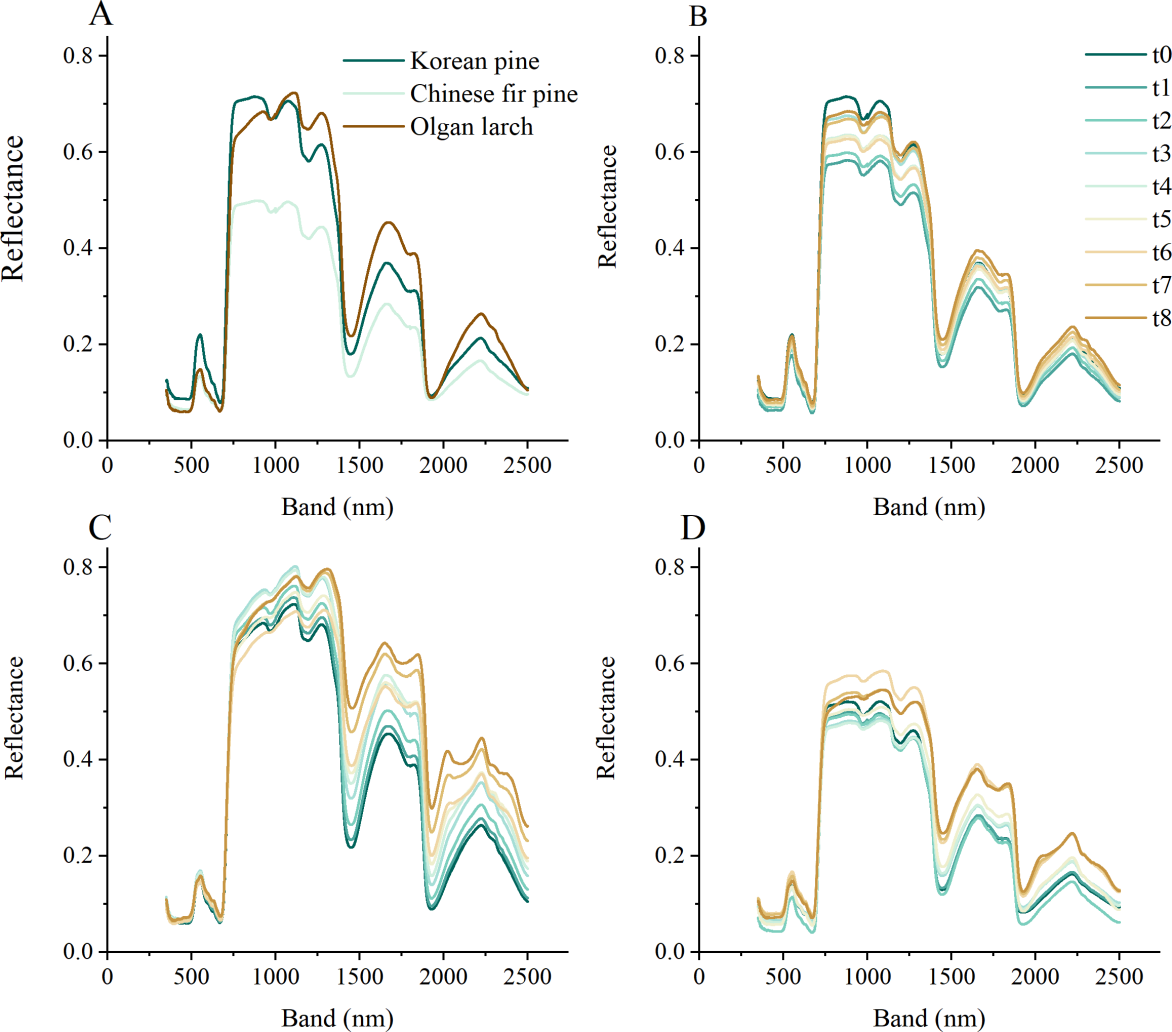
The hyperspectral reflectance of three morphologies of conifer needles. **(A)** Reflectance at the beginning time. Reflectance changes over time for Korean pine **(B)**, Olgan larch **(C)**, and Chinese fir pine **(D)**.

Different morphologies of conifer needles exhibit varying reflectance under different leaf water content (**Figure 3**). Like the leaves of broad-leaved tree species, the reflectance of needles also exhibits four peaks (560, 770-1310, 1670-1850, 2240 nm), with the second peak covering the widest bands and displaying the highest reflectivity (**Figure 3A**). Among the three tree species, Korean pine, and Olgan larch have the most similar reflectance, and Chinese fir pine has the lowest reflectance.

The reflectance of the three tree species gradually decreased over time (**Figures 3B–D**), except for the reflectance changes around 1000 nm, which showed no obvious regularity. The change in reflectance was more significant in the near infrared region (NIR, 780-1300 nm) and the SWIR region than in the VIS region. Among the three species, Olgan larch exhibited the most pronounced changes (about 0.2) in reflectance over time, while Korean pine showed the least changes (about 0.1).

### 3.3 The leaf water sensitive bands

**Figure 4:**
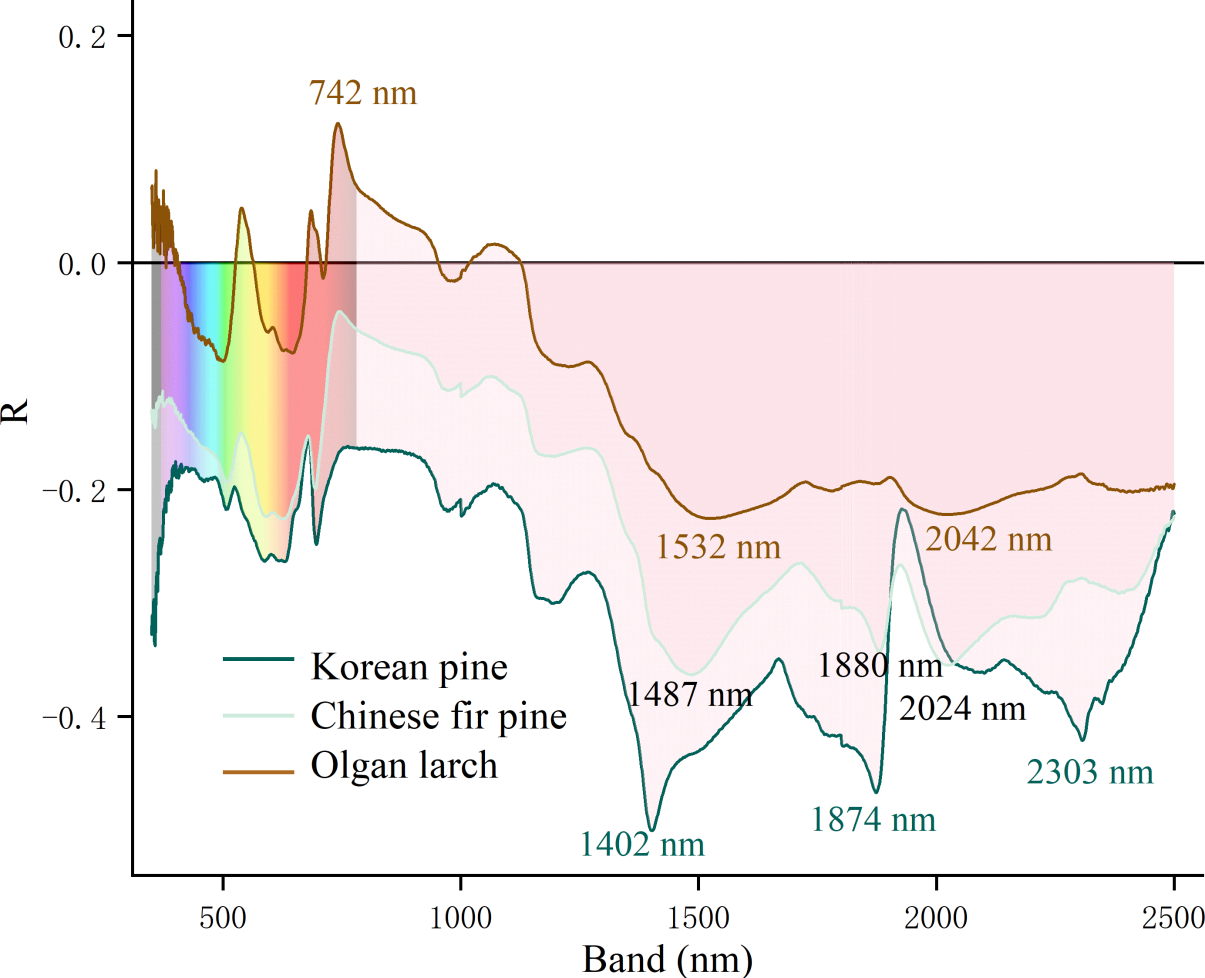
The leaf water sensitive bands of three morphologies of conifer needles. The ordinate is the Pearson correlation coefficient (R).

Water sensitive bands are mainly concentrated in the SWIR region, and exhibit negative correlations (**Figure 4**). Different morphologies of conifer needles have distinct characteristics. For Korean pine, the three most relevant bands are 1402, 1874, and 2303 nm. However, for Chinese fir pine, they are 1487, 1880, and 2024 nm. For Olgan larch, there is a most positive band at 742 nm. The changes of R values are less pronounced in the SWIR region compared to the other two morphologies of conifer needles.

### 3.4 The applicability of the commonly used water indices

Twelve water indices were used to estimate the water content of three morphologies of conifer needles. The estimation results of different water indices showed varying distribution intervals (**Supplementary Figure S1**), ranging from -0.5 to 3.5. Among the 12 water indices, three water indices (MSI-1, MSI-2, and DDI) showed a negative correlation, while the other nine showed a positive correlation. The calculation results of the water index LWI were the largest, even several times larger than the other indices. Some water indices showed similar results, such as WI and FPWBI, MSI-1 and MSI-2, NDII and GVMI, etc.

**Figure 5:**
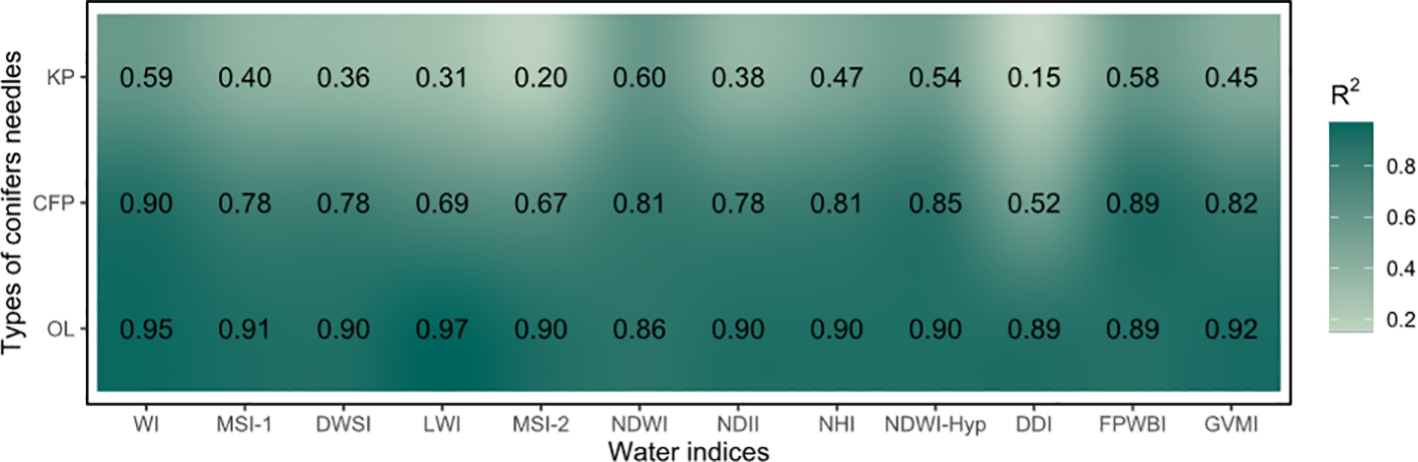
Correlation between 12 water indices and water content of three morphologies of conifer needles. KP, CFP, and OL are the abbreviations of Korean pine, Chinese fir pine, and Olgan larch, respectively. The corresponding scatter plots are shown in **Supplementary Figures S2**, **S3**, and **S4**.

The correlation results are different in various water indices and morphologies of conifer needles (**Figure 5**). The correlation was lower for Korean pine (mean value 0.42), but higher for Olgan larch (mean value 0.78) and Chinese fir pine (mean value 0.91). The R^2^ values of the WI, NDWI, NDWI-Hyp, and FPWBI indices are all above 0.50.

**Figure 6:**
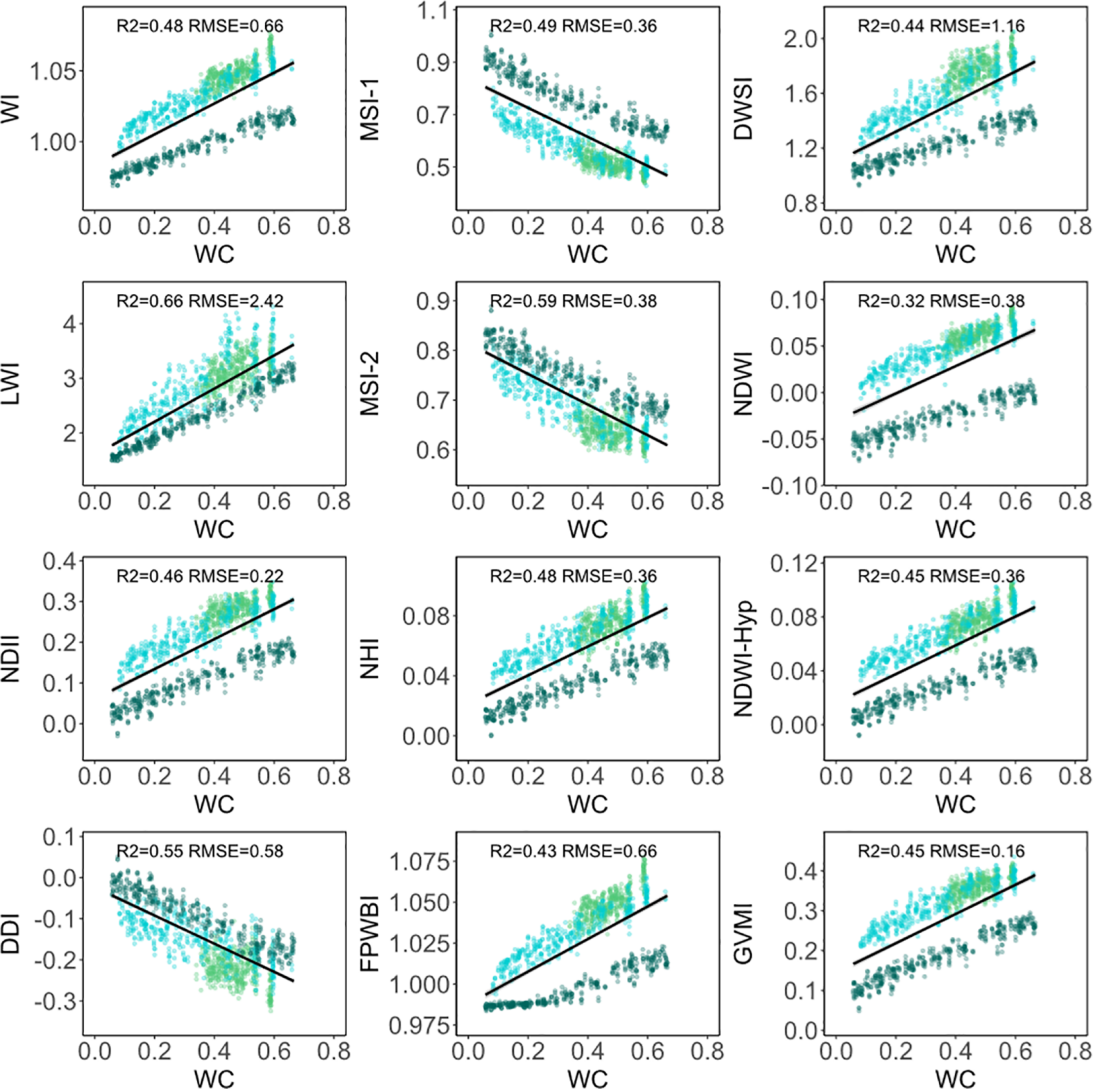
Scatter plots between 12 water indices and water content of three morphologies of conifer needles. The dark green, light blue, and light green dots represent Olgan larch, Chinese fir pine, and Korean pine, respectively.

When considering the three morphologies of conifer needles simultaneously, the correlation between 12 water indices and water content is lower than when modeling each morphology of conifer needles separately (**Figure 6**). The R^2^ values of the LWI, MSI-2, and DDI indices are all above 0.50.

### 3.5 Partial least squares regression method predictions of water content

**Figure 7:**
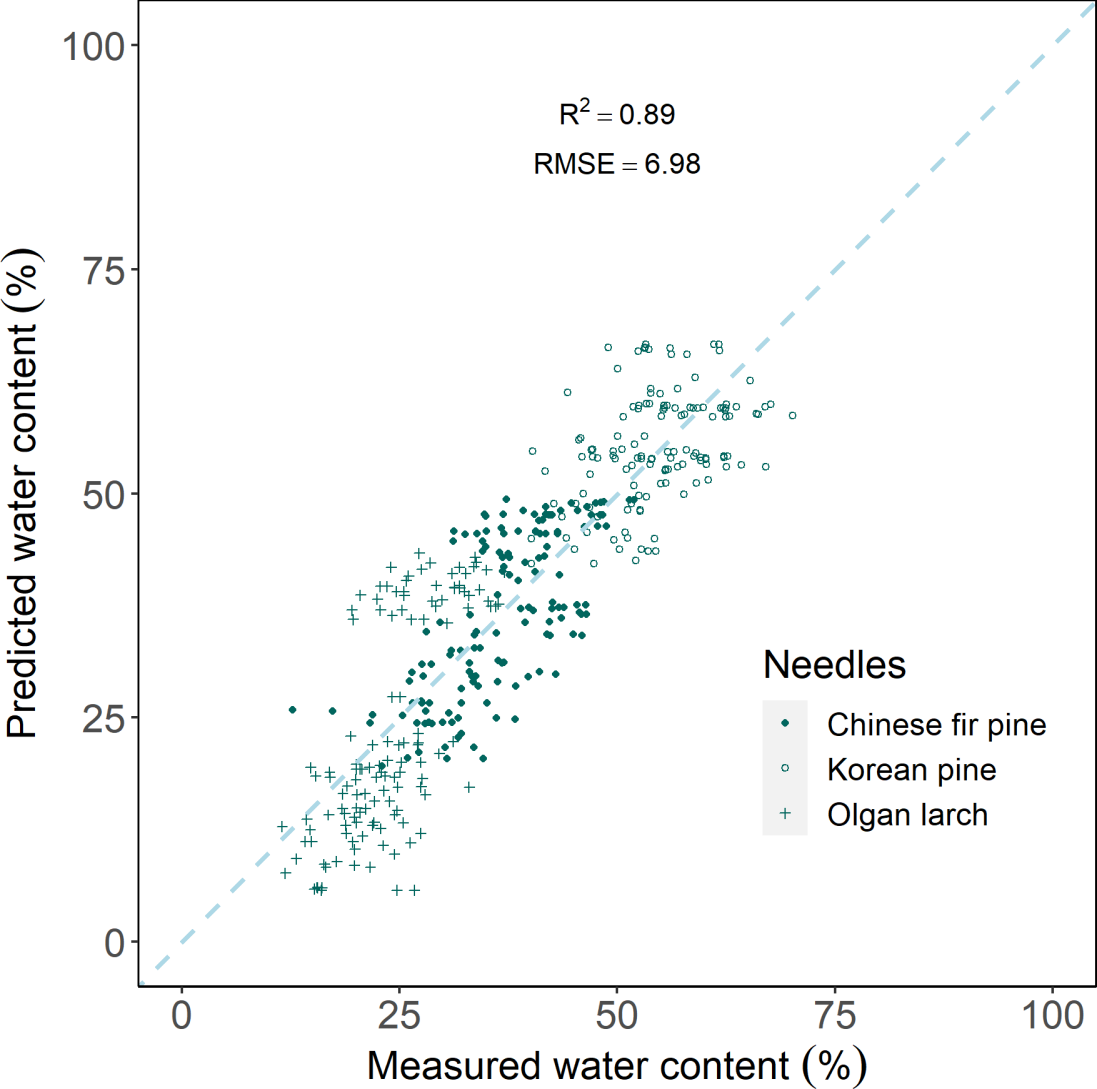
Scatter plot of measured water content and predicted water content by partial least squares regression model.

The results indicate that the leaf water content in different conifers is well-fitted. PLSR prediction, using hyperspectral reflectance, can well predict water content (R^2^ = 0.89, RMSE=6.89) for all three morphologies of conifer needles (**Figure 7**).

## 4 Discussion

In this study, we utilized hyperspectral reflectance in 12 water indices and PLSR models to assess the leaf water content of three morphologies of conifer needles. We found that the performance of different water indices varied, and to a certain extent, each water index was suitable for estimating the water content of a single type of conifer needles. The PLSR model was effective in estimating the water content of all three morphologies of conifer needles, demonstrating that the hyperspectral PLSR model is a promising tool for estimating canopy water content of forest with high species diversity on a larger scale.

### 4.1 Water loss differences

Trees with different wood properties (diffuse-porous, ring-porous, and non-porous) process different xylem structures, which is the main reason for the differences in their hydraulic efficiency (McCulloh et al., 2010). In diffuse-porous and ring-porous trees (Angiosperms), water is transported the evaporating surface through dense vein networks and vessels within the leaves. Conversely, in non-porous trees (Gymnosperms), water transport within needles depends on a single main vein, with narrow tracheids acting as the water transport cells (Hacke et al., 2015). The thick cuticle of epidermis, sunken stomata, and rounder shape of overall leaves, help prevent water loss through the needles. So the leaf hydraulic conductance of non-porous trees is the lowest (Jin and Wang, 2016). In addition, the stomatal density of Gymnosperms is lower than that of Angiosperms, which is also one of the possible reasons (Xiong and Flexas, 2020).

Three types of conifers are all non-porous trees and Pinaceae plants in our study, while the rates of water loss exhibit significantly differences. This disparity in hydraulic trait may be ascribed to variations in leaf traits and anatomy, such as leaf venation (Sack and Scoffoni, 2013), stomata (Buckley, 2005), anatomical traits of cells and tissues (John et al., 2013), and so on. Concerning leaf traits, the needles of Korean pine are the longest and triangular in section, those of Chinese fir pine are the widest and relatively hard, and the needles of Olgan larch are small and soft.

**Figure 8:**
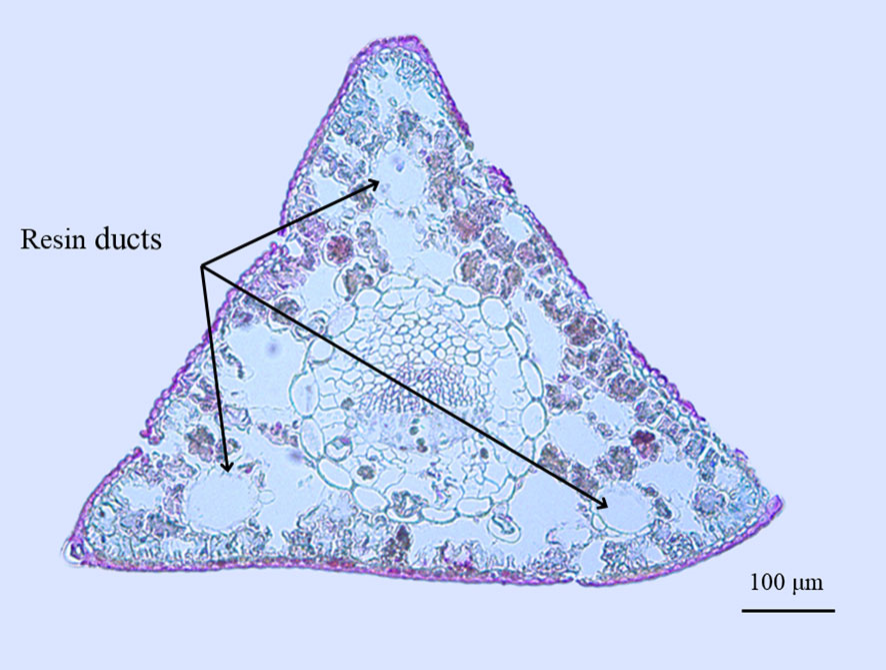
Needle cross section of Korean pine. Three resin ducts are marked. The cell was stained with safranin-fast green. The method is consistent with xylem anatomy (Zhang et al., 2020).

The anatomy of the Korean pine needles is shown in **Figure 8**. One notable feature is that stomata are only appeared on two sides, with the abaxial side (based on the position of the phloem) lacking stomata (Ghimire et al., 2015a). There are three medial resin ducts in cross section, which is more than those of the other two tree species (that typically have only one or two resin ducts). We infer that this may be one of the reasons for its slower water loss. On the other hand, the embolism-resistant xylem of the *Larix* genus (*Larix occidentalis*) is the least, when compared with that of conifers of the *Pinus* and *Pseudotsuga* genera (Miller and Johnson, 2017). The xylem is easily embolized implies that the water use strategy is not conservative, which explains why the Olgan larch needles lose water the fastest. In addition, the *Pinus* genus (*Pinus sylvestris*) has a thicker cell wall thickness and a lower average fraction of intercellular airspace compared to *Larix* genus (*Larix decidua*) (Eckert et al., 2021). Both of these characteristics provide resistance to water evaporation from the stomata, which may explain the slower water loss of *Pinus sylvestris* needles. In terms of Chinese fir pine needles, they have a continuous single-layered hypodermis (Ghimire et al., 2015b), whereas Korean pine needles are typically one to two layered. The differences in hypodermis may be one of the reasons for the variance in water loss between these two species. Lastly, another difference that cannot be ignored is the quantity of resin ducts. Does a higher quantity and proportion of resin ducts in the cross section result in slower water loss of needles? The question remains to be explored.

### 4.2 Spectral reflectance and water indices

Water has high absorption in the NIR and SWIR wavelength range, as confirmed by previous studies. Three spectral intervals (970–1150, 1330–1350, and 1584–1760 nm) have been proven to be suitable for retrieving leaf water content (Fang et al., 2017). In the study of equivalent water thickness for three mangrove trees, the spectral domain of 1125-1868 nm is found to be sensitive (Fu et al., 2024). In our recent research, we also found the SWIR region is sensitive to leaf water content (Zhang et al., 2022). The bands that make up the 12 water indices also mostly come from this interval (**Supplementary Table S1**).

Water indices can be used to promptly estimate leaf water content, which is crucial for detecting and monitoring water stress in forests (Le et al., 2023). Leaf traits or physiological indicators exert a certain degree of influence on the inversion results. In our study, the R^2^ of Korean pine is lower than the other two (**Figure 5**). We speculate that it might be attributed to the influence of leaf morphology and pigment changes, as Korean pine has lost water for an overly long period. The accuracy of water content estimation for a specific type of leaves, such as maize seedlings (Ma et al., 2016) and kiwifruit (Zhang et al., 2022), is very high. However, a challenge emerges as the inversion accuracy varies among different morphologies of leaves, even for the same water index, when the fitting coefficient is not taken into account (**Figure 6**). Consequently, separate fitting is required for different leaves, leading to a lack of generalization of the water indices.

Although the water indices have the drawback of species-specificity, they are still widely used (Zhang and Zhou, 2019; Raj et al., 2021). They provide a relatively quick and straightforward way to obtain initial estimates of parameters. Also, the existing knowledge and research basis for these water indices make them a convenient starting point for many studies. However, in the long term, more efforts should be made to develop and apply full spectra prediction models to improve the accuracy and universality of water content estimation.

### 4.3 Advantages of machine learning method

Compared with water indices, the machine learning method is more suitable for estimating the water content of multiple needles just through building one model. Methods such as PLSR (Xie et al., 2013), random forest (Xu et al., 2023), and support vector machine (Tunca et al., 2023) are considered preferable for determining water content. In recent years, state-of-the-art deep learning solutions have also been develop and attracted attention (Kamarudin et al., 2021).

In this study, we solved the problem by using PLSR, that estimation in various leaves is inconsistent (**Figure 7**). PLSR encompasses principal component analysis, canonical correlation analysis, and multiple linear regression, rendering it more appropriate for modeling multi-dimensional spectral information. Scaling the leaf-level model to the canopy level is essential for vegetation monitoring. Also, several studies have estimated the water content of the vegetation canopy based on PLSR (Mirzaie et al., 2014; Boloorani et al., 2020; Sun et al., 2021). Leaf age (Lhotakova et al., 2021) background soil reflectance (Panigrahi and Das, 2021) are both the important factors that need to be considered. This approach could be explored in future research endeavors.

### 4.4 Limitations of the study

The spectral features and relationships identified at the leaf scale cannot be directly applied to the plant canopy without modifications (Ma et al., 2019), which is an important problem in model scaling. The PROSAIL Model (PROSPECT and SAIL radiative transfer models) (Berger et al., 2018) provides a possible method to solve this problem. Parameters such as leaf pigment content, leaf area index, and average leaf inclination angle are input into the model together with leaf water content, to generate canopy reflectance data. That is, canopy reflectance data can be used to estimate water content when other parameters are determined. There has been a study on the potential applicability of the leaf radiative transfer model (PROSPECT) for Norway spruce needles (Malenovsky et al., 2006). However, new problems emerge, i.e., the large-scale acquisition and coupling of the other parameters still require further studied.

Leaf traits are influenced by leaf age and seasonal dynamics. The needles in our study were fully mature leaves during the peak growing season. However, the physiological and biochemical characteristics of leaves typically change with time during the year or more. Taking leaf age and seasonal dynamics into account is crucial for the spectral inversion of leaf traits. There are large age-related variation in leaf spectral (Chavana-Bryant et al., 2017), especially for *Cunninghamia lanceolata* (Wu et al., 2018). While for *Picea sitchensis*, the study found the reflectance of different leaf ages changes relatively little (O’Neill et al., 2002).And rapid changes were observed in the spectra in early spring and late autumn for several conifers (Hovi et al., 2017). In future studies, the effects of seasonal dynamics and leaf age should be considered and compared for different conifers.

## Data Availability

The raw data supporting the conclusions of this article will be made available by the authors, without undue reservation.

## Supplementary material

Supplementary Figure 1 Estimation results of 12 water indices under different water content. Yellow indicates negative correlation and green indicates positive correlation.

Supplementary Figure 2 Correlation scatter plots between 12 water indices (vertical ordinate) and water content (horizontal ordinate, abbreviation “WC”) of Korean pine.

Supplementary Figure 3 Correlation scatter plots between 12 water indices (vertical ordinate) and water content (horizontal ordinate, abbreviation “WC”) of Chinese fir pine.

Supplementary Figure 4 Correlation scatter plots between 12 water indices (vertical ordinate) and water content (horizontal ordinate, abbreviation “WC”) of Chinese fir pine.

Supplementary Table 1 Brief description of narrow band indices that were used in the study. Notes: BR stands for band radio; BD stands for band difference. ρ1530 stands for the spectral reflectance of band 1530 nm. min(ρ930, ρ980) stands for the minima in reflectance between 930 and 980 nm.

## References

[B1] Apan A. Held A. Phinn S. Markley J. (2004). Detecting sugarcane ‘orange rust’ disease using EO-1 Hyperion hyperspectral imagery. Int. J. Remote Sens. 25, 489–498. doi: 10.1080/01431160310001618031

[B2] Berger K. Atzberger C. Danner M. D’Urso G. Mauser W. Vuolo F.. (2018). Evaluation of the PROSAIL model capabilities for future hyperspectral model environments: A review study. Remote Sens. 10 (1), 85. doi: 10.3390/rs10010085

[B3] Boloorani A. D. Ranjbareslamloo S. Mirzaie S. Bahrami H. A. Mirzapour F. Tehrani N. A. (2020). Spectral behavior of Persian oak under compound stress of water deficit and dust storm. Int. J. Appl. Earth Obs. Geoinf. 88, 102082. doi: 10.1016/j.jag.2020.102082

[B4] Buckley T. N. (2005). The control of stomata by water balance. New Phytol. 168, 275–291. doi: 10.1111/j.1469-8137.2005.01543.x 16219068

[B5] Burnett A. C. Anderson J. Davidson K. J. Ely K. S. Lamour J. Li Q. Y.. (2021). best-practice guide to predicting plant traits from leaf-level hyperspectral data using partial least squares regression. J. Exp. Bot. 72, 6175–6189. doi: 10.1093/jxb/erab295 34131723

[B6] Ceccato P. Gobron N. Flasse S. Pinty B. Tarantola S. (2002). Designing a spectral index to estimate vegetation water content from remote sensing data: Part 1 - Theoretical approach. Remote Sens. Environ. 82, 188–197. doi: 10.1016/s0034-4257(02)00037-8

[B7] Chavana-Bryant C. Malhi Y. Wu J. Asner G. P. Anastasiou A. Enquist B. J.. (2017). Leaf aging of Amazonian canopy trees as revealed by spectral and physiochemical measurements. New Phytol. 214, 1049–1063. doi: 10.1111/nph.13853 26877108

[B8] Eckert D. Martens H. J. Gu L. Jensen A. M. (2021). CO_2_ refixation is higher in leaves of woody species with high mesophyll and stomatal resistances to CO_2_ diffusion. Tree Physiol. 41, 1450–1461. doi: 10.1093/treephys/tpab016 33595079 PMC8359682

[B9] Einzmann K. Atzberger C. Pinnel N. Glas C. Böck S. Seitz R.. (2021). Early detection of spruce vitality loss with hyperspectral data: Results of an experimental study in Bavaria, Germany. Remote Sens. Environ. 266, 112676. doi: 10.1016/j.rse.2021.112676

[B10] Einzmann K. Ng W. T. Immitzer M. Bachmann M. Pinnel N. Atzberger C. (2014). Method analysis for collecting and processing in-situ hyperspectral needle reflectance data for monitoring Norway spruce. Photogramm Fernerkun 5, 423–434. doi: 10.1127/1432-8364/2014/0234

[B11] Fang M. Ju W. Zhan W. Cheng T. Qiu F. Wang J. (2017). A new spectral similarity water index for the estimation of leaf water content from hyperspectral data of leaves. Remote Sens. Environ. 196, 13–27. doi: 10.1016/j.rse.2017.04.029

[B12] Fu B. Wu Y. Zhang S. Sun W. Jia M. Deng T.. (2024). Synergistic retrieval of mangrove vital functional traits using field hyperspectral and satellite data. Int. J. Appl. Earth Obs. Geoinf. 131, 103963. doi: 10.1016/j.jag.2024.103963

[B13] Gao B. C. (1996). NDWI - A normalized difference water index for remote sensing of vegetation liquid water from space. Remote Sens. Environ. 58, 257–266. doi: 10.1016/s0034-4257(96)00067-3

[B14] Ghimire B. Lee C. Yang J. Heo K. (2015a). Comparative leaf anatomy of native and cultivated *Pinus* (Pinaceae) in Korea: implication for the subgeneric classification. Plant Systematics Evol. 301, 531–540. doi: 10.1007/s00606-014-1090-0

[B15] Ghimire B. Lee C. Yang J. Heo K. (2015b). Comparative leaf anatomy of some species of *Abies* and *Picea* (Pinaceae). Acta Botanica Brasilica 29, 346–353. doi: 10.1590/0102-33062014abb0009

[B16] Hacke U. G. Lachenbruch B. Pittermann J. Mayr S. Domec J.-C. Schulte P. J. (2015). “The hydraulic architecture of conifers,” in Functional and Ecological Xylem Anatomy. Ed. Hacke U. (Springer International Publishing, Cham), 39–75.

[B17] Hardisky M. A. Klemas V. Smart R. M. (1983). The influence of soil-salinity, growth form, and leaf moisture on the spectral radiance of spartina-alterniflora canopies. Photogramm Eng. Rem S 49, 77–83.

[B18] Hejtmanek J. Stejskal J. Cepl J. Lhotakova Z. Korecky J. Krejzkova A.. (2022). Revealing the complex relationship among hyperspectral reflectance, photosynthetic pigments, and growth in Norway spruce ecotypes. Front. Plant Sci. 13. doi: 10.3389/fpls.2022.721064 PMC9197180 35712586

[B19] Hovi A. Raitio P. Rautiainen M. (2017). A spectral analysis of 25 boreal tree species. Silva Fennica 51, 7753. doi: 10.14214/sf.7753

[B20] Hunt E. R. Rock B. N. (1989). Detection of changes in leaf water-content using near-infrared and middle-infrared reflectances. Remote Sens. Environ. 30, 43–54. doi: 10.1016/0034-4257(89)90046-1

[B21] Jin Y. Wang C. (2016). Leaf hydraulic traits and their trade-offs for nine Chinese temperate tree species with different wood properties. Chin. J. Plant Ecol. 40, 702–710. doi: 10.17521/cjpe.2016.0064

[B22] John G. P. Scoffoni C. Sack L. (2013). ALLOMETRY OF CELLS AND TISSUES WITHIN LEAVES. Am. J. Bot. 100, 1936–1948. doi: 10.3732/ajb.1200608 24070860

[B23] Kamarudin M. H. Ismail Z. H. Saidi N. B. (2021). Deep learning sensor fusion in plant water stress assessment: a comprehensive review. Appl. Sci-Basel 11, 1403. doi: 10.3390/app11041403

[B24] Khan A. Vibhute A. D. Mali S. Patil C. H. (2022). A systematic review on hyperspectral imaging technology with a machine and deep learning methodology for agricultural applications. Ecol. Inform 69, 101678. doi: 10.1016/j.ecoinf.2022.101678

[B25] Lawlor D. W. (2002). Limitation to photosynthesis in water-stressed leaves: Stomata vs. metabolism and the role of ATP. Ann. Bot. 89, 871–885. doi: 10.1093/aob/mcf110 12102513 PMC4233810

[B26] Le T. S. Harper R. Dell B. (2023). Application of remote sensing in detecting and monitoring water stress in forests. Remote Sens. 15, 3360. doi: 10.3390/rs15133360

[B27] Lhotakova Z. Kopackova-Strnadova V. Oulehle F. Homolova L. Neuwirthova E. Svik M.. (2021). Foliage biophysical trait prediction from laboratory spectra in Norway spruce is more affected by needle age than by site soil conditions. Remote Sens. 13, 391. doi: 10.3390/rs13030391

[B28] Ling B. Goodin D. G. Raynor E. J. Joern A. (2019). Hyperspectral analysis of leaf pigments and nutritional elements in tallgrass prairie vegetation. Front. Plant Sci. 10. doi: 10.3389/fpls.2019.00142 PMC6397892 30858853

[B29] Liu N. Hokanson E. W. Hansen N. Townsend P. A. (2023). Multi-year hyperspectral remote sensing of a comprehensive set of crop foliar nutrients in cranberries. Isprs J. Photogrammetry Remote Sens. 205, 135–146. doi: 10.1016/j.isprsjprs.2023.10.003

[B30] Ma B. Xu A. Zhang S. Wu L. Ieee (2016). “Retrieval of leaf water content for maize seedlings in visible near infrared and thermal infrared spectra,” in Proceedings of the 36th IEEE International Geoscience and Remote Sensing Symposium (IGARSS), Beijing, PEOPLES R CHINA, 2016 Jul 10-15. 6930–6933.

[B31] Ma S. Zhou Y. Gowda P. H. Dong J. Zhang G. Kakani V. G.. (2019). Application of the water-related spectral reflectance indices: A review. Ecol. Indic. 98, 68–79. doi: 10.1016/j.ecolind.2018.10.049

[B32] Malenovsky Z. Albrechtová J. Lhotáková Z. Zurita-Milla R. Clevers J. Schaepman M. E.. (2006). Applicability of the PROSPECT model for Norway spruce needles. Int. J. Remote Sens. 27, 5315–5340. doi: 10.1080/01431160600762990

[B33] McCulloh K. Sperry J. S. Lachenbruch B. Meinzer F. C. Reich P. B. Voelker S. (2010). Moving water well: comparing hydraulic efficiency in twigs and trunks of coniferous, ring-porous, and diffuse-porous saplings from temperate and tropical forests. New Phytol. 186, 439–450. doi: 10.1111/j.1469-8137.2010.03181.x 20158616

[B34] Mevik B.-H. Wehrens R. (2007). The pls package: Principal component and partial least squares regression in R. J. Stat. Software 18, 1–23. doi: 10.18637/jss.v018.i02

[B35] Miller M. L. Johnson D. M. (2017). Vascular development in very young conifer seedlings: Theoretical hydraulic capacities and potential resistance to embolism. Am. J. Bot. 104, 979–992. doi: 10.3732/ajb.1700161 28724592

[B36] Mirzaie M. Darvishzadeh R. Shakiba A. Matkan A. A. Atzberger C. Skidmore A. (2014). Comparative analysis of different uni- and multi-variate methods for estimation of vegetation water content using hyper-spectral measurements. Int. J. Appl. Earth Observation Geoinformation 26, 1–11. doi: 10.1016/j.jag.2013.04.004

[B37] Naik B. B. Naveen H. R. Sreenivas G. Choudary K. K. Devkumar D. Adinarayana J. (2020). Identification of water and nitrogen stress indicative spectral bands using hyperspectral remote sensing in maize during post-monsoon season. J. Indian Soc. Remote 48, 1787–1795. doi: 10.1007/s12524-020-01200-w

[B38] O’Neill A. L. Kupiec J. A. Curran P. J. (2002). Biochemical and reflectance variation throughout a Sitka spruce canopy. Remote Sens. Environ. 80, 134–142. doi: 10.1016/s0034-4257(01)00294-2

[B39] Panigrahi N. Das B. S. (2021). Evaluation of regression algorithms for estimating leaf area index and canopy water content from water stressed rice canopy reflectance. Inf. Process. Agric. 8, 284–298. doi: 10.1016/j.inpa.2020.06.002

[B40] Penuelas J. Pinol J. Ogaya R. Filella I. (1997). Estimation of plant water concentration by the reflectance water index WI (R900/R970). Int. J. Remote Sens. 18, 2869–2875. doi: 10.1080/014311697217396

[B41] Pimstein A. Eitel J. U. H. Long D. S. Mufradi I. Karnieli A. Bonfil D. J. (2009). A spectral index to monitor the head-emergence of wheat in semi-arid conditions. Field Crops Res. 111, 218–225. doi: 10.1016/j.fcr.2008.12.009

[B42] Quemada C. Perez-Escudero J. M. Gonzalo R. Ederra I. Santesteban L. G. Torres N.. (2021). Remote sensing for plant water content monitoring: A review. Remote Sens. 13, 2088. doi: 10.3390/rs13112088

[B43] Raj R. Walker J. P. Pingale R. Banoth B. N. Jagarlapudi A. (2021). Leaf nitrogen content estimation using top-of-canopy airborne hyperspectral data. Int. J. Appl. Earth Obs. Geoinf. 104, 102584. doi: 10.1016/j.jag.2021.102584

[B44] Rock B. N. Vogelmann J. E. Williams D. L. Vogelmann A. F. Hoshizaki T. (1986). Remote detection of forest damage. Bioscience 36, 439–445. doi: 10.2307/1310339

[B45] Sack L. Scoffoni C. (2013). Leaf venation: structure, function, development, evolution, ecology and applications in the past, present and future. New Phytol. 198, 983–1000. doi: 10.1111/nph.12253 23600478

[B46] Seelig H. D. Hoehn A. Stodieck L. S. Klaus D. M. Adams W. W. Emery W. J. (2008). The assessment of leaf water content using leaf reflectance ratios in the visible, near-, and short-wave-infrared. Int. J. Remote Sens. 29, 3701–3713. doi: 10.1080/01431160701772500

[B47] Serbin S. P. Townsend P. A. (2020). “Scaling functional traits from leaves to canopies,” in Remote Sensing of Plant Biodiversity. Eds. Cavender-Bares J. Gamon J. A. Townsend P. A. (Springer International Publishing, Cham), 43–82.

[B48] Stimson H. C. Breshears D. D. Ustin S. L. Kefauver S. C. (2005). Spectral sensing of foliar water conditions in two co-occurring conifer species:: *Pinus edulis* and *Juniperus monosperma* . Remote Sens. Environ. 96, 108–118. doi: 10.1016/j.rse.2004.12.007

[B49] Strachan I. B. Pattey E. Boisvert J. B. (2002). Impact of nitrogen and environmental conditions on corn as detected by hyperspectral reflectance. Remote Sens. Environ. 80, 213–224. doi: 10.1016/s0034-4257(01)00299-1

[B50] Sun H. Feng M. Xiao L. Yang W. Ding G. Wang C.. (2021). Potential of multivariate statistical technique based on the effective spectra bands to estimate the plant water content of wheat under different irrigation regimes. Front. Plant Sci. 12. doi: 10.3389/fpls.2021.631573 PMC7952645 33719305

[B51] Tunca E. Koksal E. S. Ozturk E. Akay H. Taner S. C. (2023). Accurate estimation of sorghum crop water content under different water stress levels using machine learning and hyperspectral data. Environ. Monit Assess. 195, 877. doi: 10.1007/s10661-023-11536-8 37353582

[B52] Ustin S. L. Roberts D. A. Gardner M. Dennison P. Ieee (2002). “Evaluation of the potential of Hyperion data to estimate wildfire hazard in the Santa Ynez Front Range, Santa Barbara, California,” in Proceedings of the IEEE International Geoscience and Remote Sensing Symposium (IGARSS 2002)/24th Canadian Symposium on Remote Sensing, Toronto, Canada, Jun 24-28. 796–798.

[B53] Verrelst J. Malenovsky Z. van der Tol C. Camps-Valls G. Gastellu-Etchegorry J. P. Lewis P.. (2019). Quantifying vegetation biophysical variables from imaging spectroscopy data: A review on retrieval methods. Surveys Geophysics 40, 589–629. doi: 10.1007/s10712-018-9478-y PMC7613341 36081834

[B54] Wang Q. Li P. H. (2012). Hyperspectral indices for estimating leaf biochemical properties in temperate deciduous forests: Comparison of simulated and measured reflectance data sets. Ecol. Indic. 14, 56–65. doi: 10.1016/j.ecolind.2011.08.021

[B55] Wang Z. Huang H. Wang H. Penuelas J. Sardans J. Niinemets U.. (2022). Leaf water content contributes to global leaf trait relationships. Nat. Commun. 13, 5525. doi: 10.1038/s41467-022-32784-1 36130948 PMC9492732

[B56] Wu Q. Song C. Song J. Wang J. Chen S. Yu B. (2018). Impacts of leaf age on canopy spectral signature variation in evergreen Chinese fir forests. Remote Sens. 10, 262. doi: 10.3390/rs10020262

[B57] Xie C. Q. Li X. L. Nie P. C. He Y. (2013). Application of time series hyperspectral imaging (ts-hsi) for determining water content within tea leaves during drying. T Asabe 56, 1431–1440. doi: 10.1007/s11947-012-0928-0

[B58] Xiong D. Flexas J. (2020). From one side to two sides: the effects of stomatal distribution on photosynthesis. New Phytol. 228, 1754–1766. doi: 10.1111/nph.16801 32652573

[B59] Xiong D. Nadal M. (2020). Linking water relations and hydraulics with photosynthesis. Plant J. 101, 800–815. doi: 10.1111/tpj.14595 31677190

[B60] Xu Z. H. Li B. Yu H. Zhang H. F. Guo X. Y. Li Z. L.. (2023). Changing Relationships between Water Content and Spectral Features in Moso Bamboo Leaves under Pantana phyllostachysae Chao Stress. Forests 14, 702. doi: 10.3390/f14040702

[B61] Yuan D. Zhu L. Cherubini P. Li Z. Zhang Y. Wang X. (2021). Species-specific indication of 13 tree species growth on climate warming in temperate forest community of northeast China. Ecol. Indic. 133, 108389. doi: 10.1016/j.ecolind.2021.108389

[B62] Zhang Z. Fu Y. Li H. Guo J. Pan Y. Zhang Y.. (2022). Monitoring the leaf equivalent water thickness of kiwifruit in high temperature using leaf spectral reflectance. Spectrosc Lett. 55, 659–672. doi: 10.1080/00387010.2022.2149558

[B63] Zhang H. McDowell N. G. Adams H. D. Wang A. Wu J. Jin C.. (2020). Divergences in hydraulic conductance and anatomical traits of stems and leaves in three temperate tree species coping with drought, N addition and their interactions. Tree Physiol. 40, 230–244. doi: 10.1093/treephys/tpz135 31860728

[B64] Zhang Y. Wu J. Wang A. (2022). Comparison of various approaches for estimating leaf water content and stomatal conductance in different plant species using hyperspectral data. Ecol. Indic. 142, 109278. doi: 10.1016/j.ecolind.2022.109278

[B65] Zhang F. Zhou G. (2019). Estimation of vegetation water content using hyperspectral vegetation indices: a comparison of crop water indicators in response to water stress treatments for summer maize. BMC Ecol. 19, 18. doi: 10.1186/s12898-019-0233-0 31035986 PMC6489241

[B66] Zlobin I. E. (2022). Linking the growth patterns of coniferous species with their performance under climate aridization. Sci. Total Environ. 831, 154971. doi: 10.1016/j.scitotenv.2022.154971 35367548

