# Supplementary material for: Water content estimation of conifer needles using leaf-level hyperspectral data
Source: Front Plant Sci. 2024 Sep 6;15:1428212. doi: 10.3389/fpls.2024.1428212 (PMC11412879; doi:10.3389/fpls.2024.1428212)
Supplement: Supplementary Figure 1 — Estimation results of 12 water indices under different water content. Yellow indicates negative correlation and green indicates positive correlation. [file DataSheet1.docx]

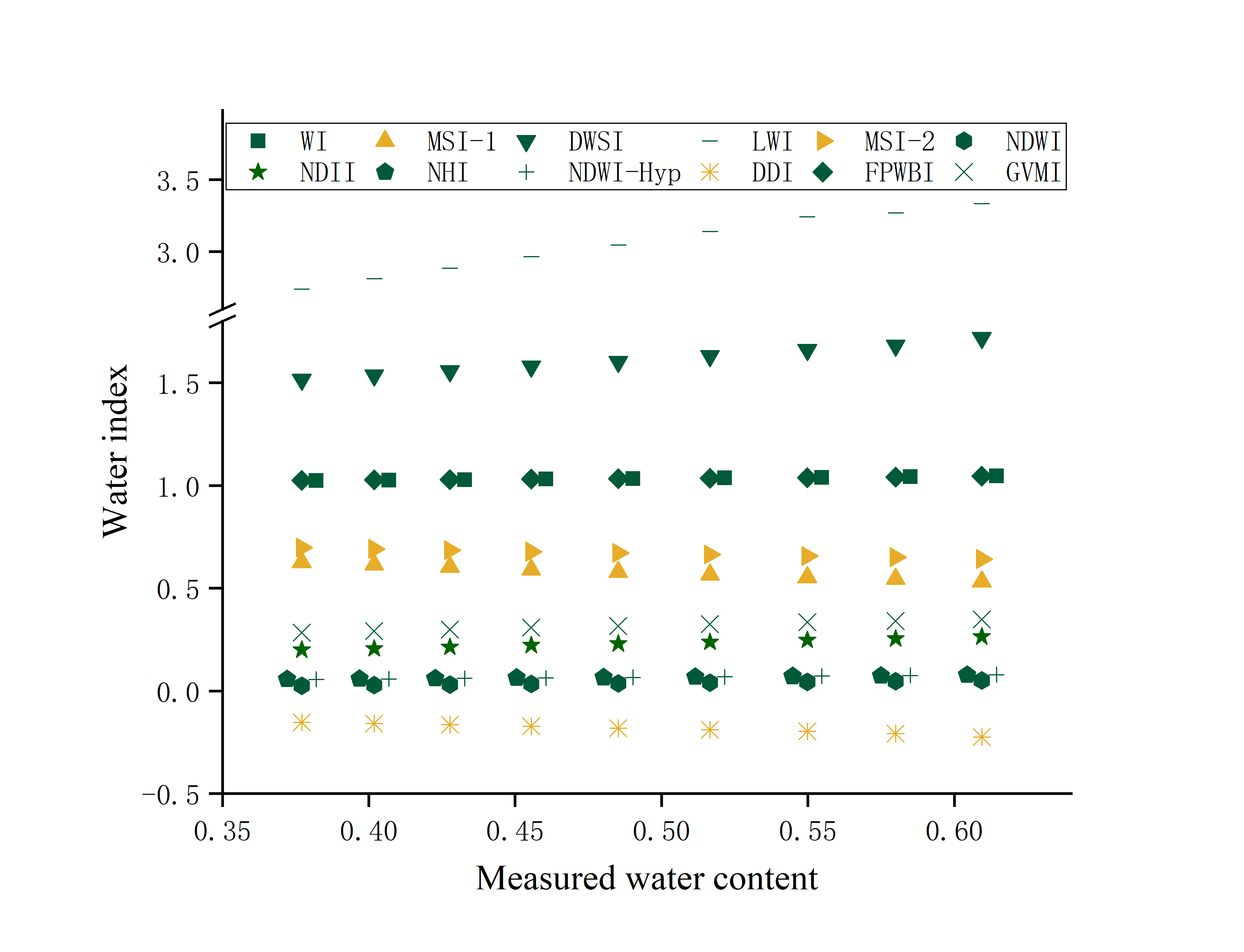


Fig. S1. Estimation results of 12 water indices under different water content.

Yellow indicates negative correlation and green indicates positive correlation.


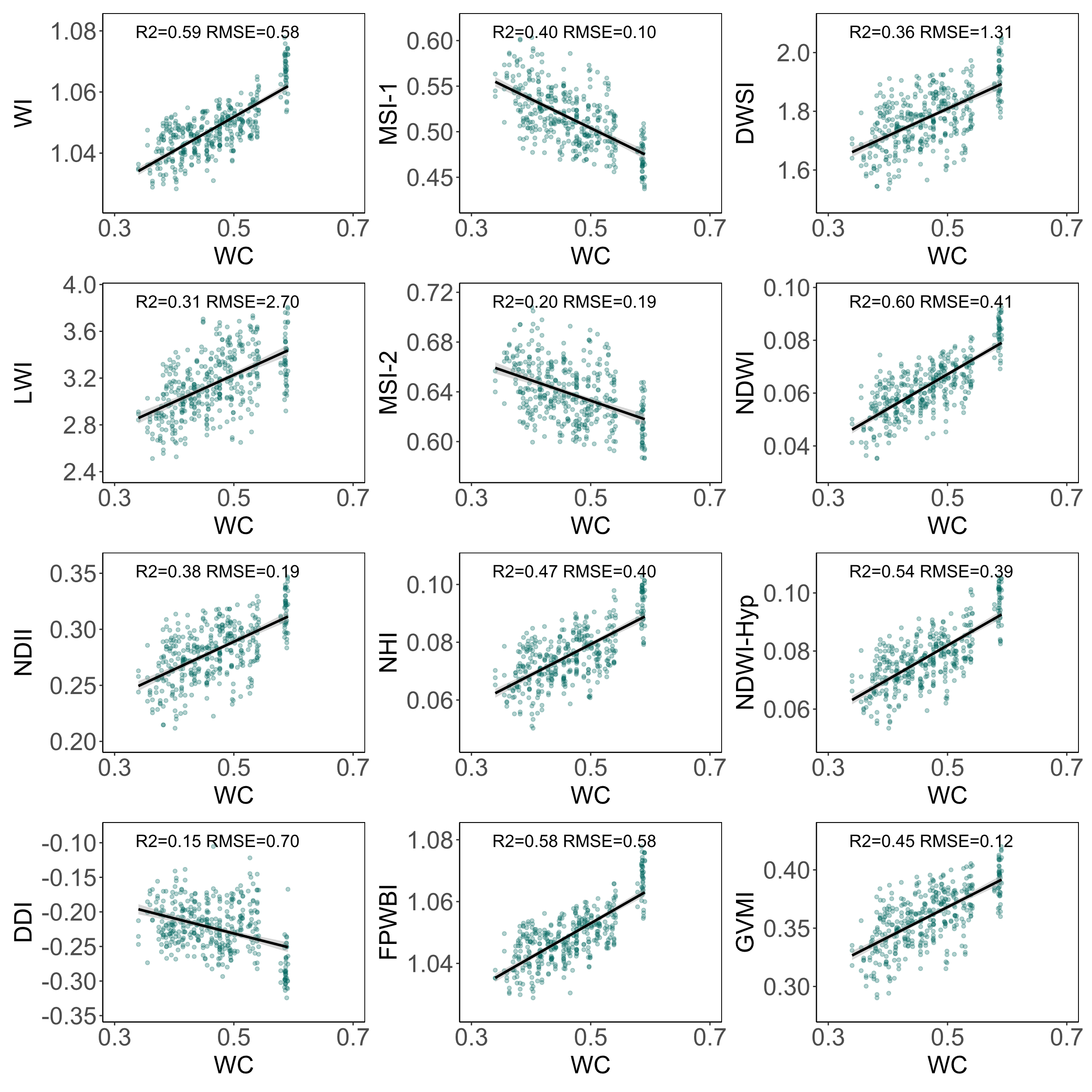


Fig. S2. Correlation scatter plots between 12 water indices (vertical ordinate) and water content (horizontal ordinate, abbreviation “WC”) of Korean pine.


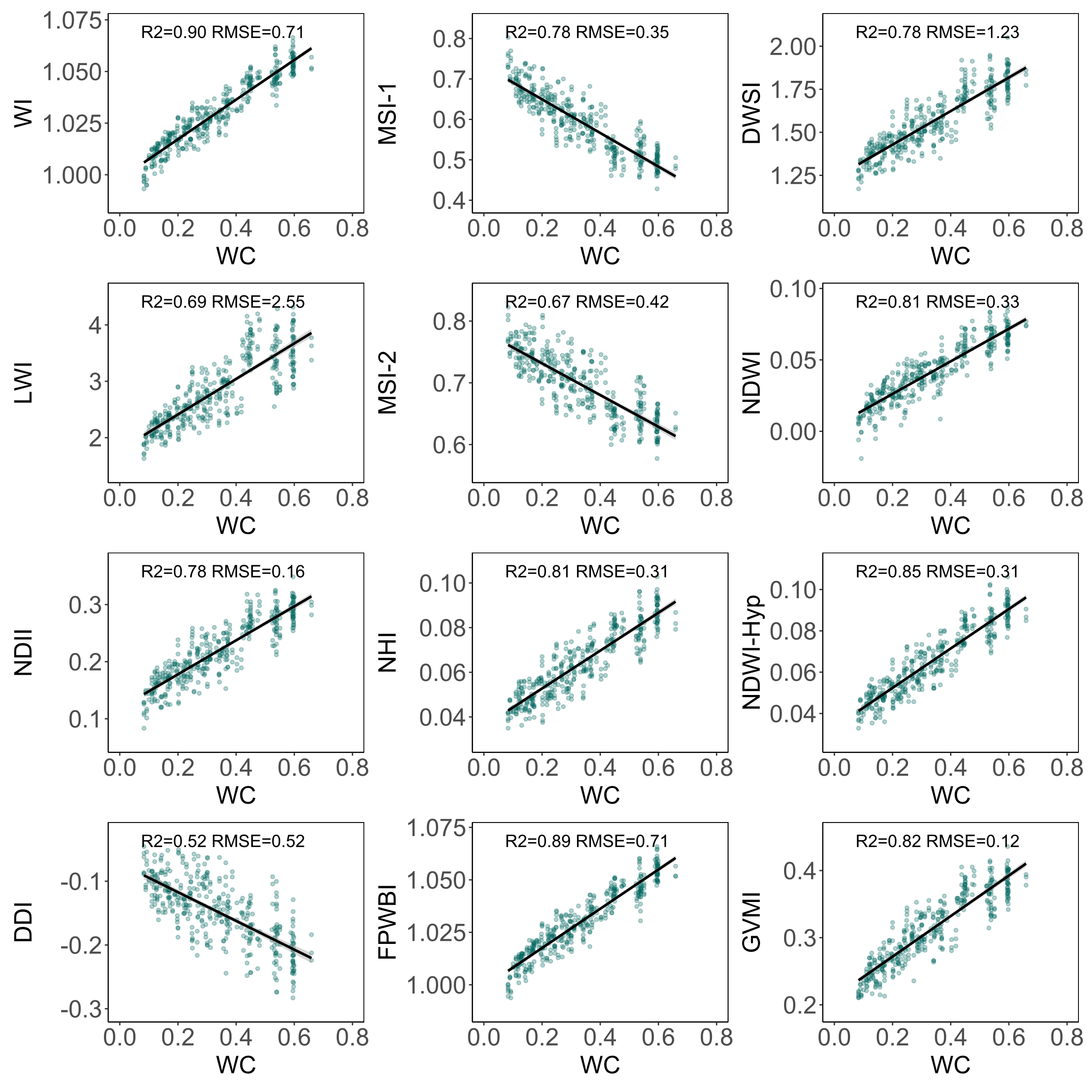


Fig. S3. Correlation scatter plots between 12 water indices (vertical ordinate) and water content (horizontal ordinate, abbreviation “WC”) of Chinese fir pine.


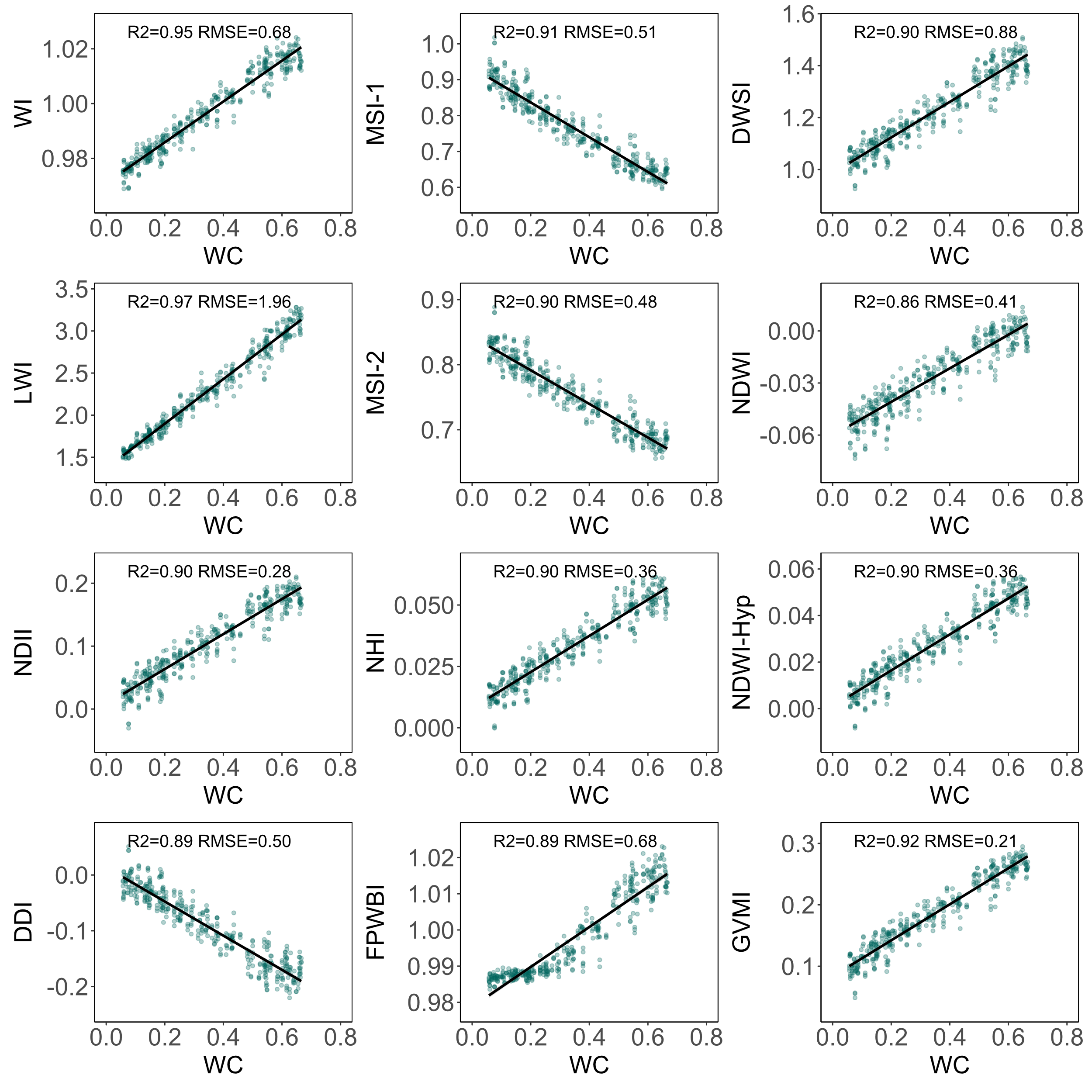


Fig. S4. Correlation scatter plots between 12 water indices (vertical ordinate) and water content (horizontal ordinate, abbreviation “WC”) of Chinese fir pine.

Table S1. Brief description of narrow band indices that were used in the study.

| **Num** | **Index** | **Index name** | **Equation** | **Related to** |
| --- | --- | --- | --- | --- |
| 1 | WI | Water Index | BR(900,970) | plant water content |
| 2 | MSI-1 | Moisture Stress Index | BR(1600,820) | leaf relative water content |
| 3 | DWSI | Disease-Water Stress Index | BR(800,1660) | canopy orange rust disease |
| 4 | LWI | Leaf Water Index | BR(1300,1450) | leaf relative water content |
| 5 | MSI-2 | Moisture Stress Index | BR(1650,1230) | canopy water stress |
| 6 | NDWI | Normalized Difference Water Index | BD(860,1240) | vegetation liquid water |
| 7 | NDII | Normalized Difference Infrared Index | BD(820,1650) | Leaf water content |
| 8 | NHI | Normalized Heading Index | BD(1100,1200) | canopy relative water content |
| 9 | NDWI-Hyp | NDWI-Hyperion | BD(1070,1200) | canopy water content |
| 10 | DDI | Double Difference Index | 2ρ1530 −ρ1005 −ρ2005 | leaf equivalent water thickness |
| 11 | FPWBI | Floating Position Water Band Index | ρ900/min(ρ930, ρ980) | canopy water stresses |
| 12 | GVMI | Global Vegetation Moisture Index | ((ρ820 + 0.1) − (ρ1600 + 0.02))/  ((ρ820 + 0.1) + (ρ1600 + 0.02)) | canopy equivalent water thickness |

Notes: BR stands for band radio; BD stands for band difference.

ρ1530 stands for the spectral reflectance of band 1530 nm. min(ρ930, ρ980) stands for the minima in reflectance between 930 and 980 nm.
